# Unintentional drowning fatalities in Sweden between 2002 and 2021

**DOI:** 10.1186/s12889-024-20687-3

**Published:** 2024-11-16

**Authors:** Alexander Tyr, Emma Molander, Björn Bäckström, Andreas Claesson, Brita Zilg

**Affiliations:** 1 Swedish National Board of Forensic Medicine, Stockholm, Sweden; 2 Department of Oncology-Pathology, Karolinska Institutet, Stockholm, Sweden; 3 Swedish National Board of Forensic Medicine, Umeå, Sweden; 4 Department of Community Medicine and Rehabilitation/Forensic Medicine, Umeå University, Umeå, Sweden; 5 Department of Clinical Science and Education, Centre for Resuscitation Science, Karolinska Institutet, Södersjukhuset, Stockholm, Sweden

**Keywords:** Demographic, Ethnicity, Forensic Medicine, Epidemiology, Drowning prevention, Public Health

## Abstract

**Background:**

Despite declining over the past three decades, unintentional drownings still account for an estimated 236 000 annual deaths worldwide. Susceptibility persists amongst demographic groups and is influenced by sex, age, and socio-economic status, emphasizing the need for targeted interventions. Due to rapidly evolving population dynamics, particularly within Europe, there is a further responsibility to understand the impact of ethnicity on the risks of drowning to guide prevention.

**Methods:**

We conducted a national population-based retrospective study using data from the Swedish National Board of Forensic Medicine and Statistics Sweden for the years 2002 to 2021. The analysis includes variables such as age, sex, presence of alcohol and narcotics as well as activity undertaken at the time of drowning and type of water body. Furthermore, we considered ethnicity to identify subpopulations at greater risks.

**Results:**

Results revealed a plateau in unintentional drowning rates in Sweden since 2012, despite an overall decrease from 2002 to 2021. Findings confirm the trend that males are overrepresented within drowning statistics across all age groups, and that individuals aged > 50 constitute over half of all unintentional drownings. Men aged between 40-69 years boating, and individuals of non-Swedish origin, particularly those < 20 years of age, face a notably greater risk of drowning, underscoring the need for subpopulation-targeted prevention strategies.

**Conclusion:**

The ten-year plateau in unintentional drowning signals the need for an official national prevention strategy with annual evaluations. Suggestions also include improved parental supervision of children, further avoidance of alcohol while swimming and boating, as well as targeted swimming lessons and water competency training for individuals of non-Swedish origin.

**Supplementary Information:**

The online version contains supplementary material available at 10.1186/s12889-024-20687-3.

## Introduction

It is well established that drowning is a prominent cause of death globally. Although underreported, owing to factors such as the lack of standardized reporting systems [1], unintentional drowning deaths are estimated to account for approximately 236 000 cases annually [2]. Despite declining over the past thirty years [3] due to public health campaigns, development in infrastructure, strengthened legislation and changes in social attitudes, a large number still remain preventable, highlighting the need for more targeted interventions [4].

Whilst drowning can affect anyone, distinct demographic cohorts exhibit greater vulnerability compared to others [5]. This includes differences in sex, age, socio-economic class, and ethnicity [3]. For instance, men are disproportionately represented across all ages in the drowning literature and face a greater risk of unintentional drowning compared to females [6]. Children and adolescents carry a greater risk of drowning compared to adults as do those associated with lower socio-economic status than higher [6–9]. Evidently, it is crucial to explore prevalence rates to identify underlying factors such as age, sex, presence of alcohol or drugs and pre-drowning activity that increases drowning susceptibility in differing subpopulations.

Between 2002 to 2021, Sweden's population grew from 9 million to 10.5 million, with the proportion of non-Swedish origin individuals increasing from 15 to 26% (Statistics Sweden (SCB)). In light of such recent waves of migration and evolving population dynamics, medical professionals and lifesaving personnel have noted that individuals of non-Swedish origin, particularly children and young adults, may carry a greater risk of drowning than individuals with Swedish origin. Nevertheless, these assertions remain unconfirmed.

Previous investigations into unintentional drowning death rates in American populations have demonstrated racial disparities [8, 10–12], but less data on ethnic and cultural differences has been published, particularly within European populations [13]. Research from the Netherlands shows that ethnic minorities face a greater risk of unintentional drowning compared to native Dutch populations [14, 15] with similar findings noted in Sweden, indicating that individuals under 17 years with non-Swedish origin face a greater risk of unintentional drowning compared to Swedish counterparts [16]. Together, the literature implicated already a decade ago a need to further explore how ethnicity and increased drowning risks are linked, to tackle challenges arising from demographic shifts.

Few studies have investigated the occurrence of drowning fatalities in Sweden on a national scale. Ahlm et al. [17], examined drowning incidents in Sweden between 1992 and 2009, highlighting that middle-aged men between 50–69 are most susceptible to drowning. Claesson et al. [18] extended their analysis to fatal and non-fatal incidents spanning the years 2003–2017. Research revealed an annual average of 441 cases, with an approximately equal distribution between fatalities and injuries, but also demonstrated a marginal increase in fatal unintentional drownings between 2010–2017, further supporting the need for renewed and organized intervention strategies. Interestingly, similar trends have been reported within Dutch populations, where unintentional drowning rates also increased between 2012–2017 [15]. It may be stipulated that other European countries are exhibiting comparable inclinations.

The Swedish Civil Contingencies Agency (MSB) is responsible for overseeing public drowning statistics and awareness campaigns in Sweden, collaborating with the Swedish Lifesaving Society (SLS) for data collection. SLS is a non-profit organization that educates swimming instructors and lifeguards in addition to promoting water safety across the country. Although the Swedish government does not currently have an established official national drowning prevention strategy, it has over a number of years introduced legislation aimed at reducing unintentional drownings. This includes promoting swimming competencies in schools starting at age 12, banning alcohol consumption while boating, introduction of drivers’ licenses for small water crafts and mandating fencing and other regulations for infrastructure near water. In an attempt to further organize and outline preventative actions, the SLS formalized in 2021 a national plan in line with the United Nations (UN) global drowning resolution (A/RES/75/273). These initiatives aim to reduce accidental drownings in Sweden by at least 25% by 2033. However, evaluating the effectiveness of such preventative measures is challenging.

SLS gathers national drowning statistics by monitoring media, local community and regional reports, in addition to data from the Swedish National Board of Health and Welfare's Cause of Death Registry (CoDR). Although the CoDR provides statistics according to the International Classification of Disease (ICD), data is not scientifically reviewed and does not include details regarding unnatural deaths. Such information is instead registered and managed by the National Board of Forensic Medicine (NBFM), responsible for conducting forensic autopsies on all unnatural deaths in Sweden, including drownings.

In this nation-wide retrospective epidemiological study, we utilize data from the NBFM and SCB spanning the years 2002 to 2021 to answer three main questions:

- How have unintentional drowning rates in Sweden changed between 2002 and 2021?
- What demographic groups are overrepresented in unintentional drowning incidents in Sweden?
- Which subpopulations carry an increased risk of unintentional drowning in Sweden?

Our analysis goes beyond traditional factors such as age, sex, the presence of alcohol and narcotics in drowning victims as we also delve into additional variables, including activity undertaken at the time of drowning, type of water body involved and region of origin. We seek to identify populations and demographic cohorts at risk, to inform future preventative strategies.

## Methods

### Database extraction

All medico-legal autopsies that included drowning as terminal, underlying or contributory cause of death between 1 January 2002 and 31 December 2021 were extracted from the NBFM database (*n* = 4489). Homicides (*n* = 23), cases with place of death outside of Sweden (*n* = 32), and cases that had been autopsied in 2002, with a date of death prior to 2002 were excluded (*n* = 12). Cases incorrectly diagnosed as drownings by ICD code-9 (9th revision) were also excluded (*n* = 2).

The NBFM database includes information on age, sex, circumstances surrounding death, as well as cause and manner of death. Circumstantial information, such as type of body of water (e.g. sea, lake, river, pool) and pre-drowning activity were assessed from police records. The presence of alcohol and narcotics was collected for cases between 2002–2021. All unnatural deaths (including drowning) occurring in Swedish territory, are subject to forensic autopsy and include toxicological screening for alcohol, pharmaceutical and illicit drugs. Analyses were performed using headspace gas chromatography. All cases where blood alcohol content (BAC) was > 0.2 g/l in femoral blood, the drunk-driving limit in Sweden, were considered positive for alcohol consumption. Cases positive for narcotic classified substances in Sweden were considered positive regardless of concentrations.

Region of origin for each deceased individual was obtained from SCB, the Swedish authority responsible for producing official statistics for decision-making, debate and research. Regions were categorized according to Fig. 3C. Persons of Swedish origin were defined as those born in Sweden with at least one parent also born in Sweden.

### Statistics

All data was processed in Microsoft Excel (2019) version 1808 and presented as descriptive statistics, in terms of frequencies and ratios. Mortality rates were calculated per 100 000 inhabitants with population data for each year. Five cases, where age was not available, were not included in analyses where age was a variable.

For trend analysis of the total period 2002–2021, single linear regression analysis has been used together with Sieve-bootstrap Student’s t test [19] for a linear trend in R (R Core Team, Vienna, Austria (2023) https://www.R-project.org/ version 4.3.1). For all calculations, *p* < 0.05 was considered statistically significant. To determine whether there were multiple trends with breakpoints, piecewise regression [20] was also used in R. Single linear regression together with Sieve-boostrap Student’s t test analysis was also conducted on alcohol consumption between 2002–2021 (R, version 4.3.1).

To assess the risk of drowning for individuals based on their Swedish or non-Swedish origin, we normalized populations and expressed the risk of drowning as an odds ratio, with 1 representing an equivalent risk between Swedish and non-Swedish individuals. A ratio > 1 indicated a higher drowning risk for non-Swedish origins, whilst a ratio < 1 presented a higher risk of drowning for individuals with Swedish origin.

Data on categorical variables including, sex, age groups, manner of death, body of water and circumstances of death were presented as amount and proportions. Age groups were categorized as intervals. Manner of death was categorized as unintentional, suicide and undetermined. Body of water was categorized as sea, lake, stream or river, ice related, public pool, bathtub, pool/hot tub and other. The group “other” includes drownings that occurred in other places such as ditches, ponds and wells. Circumstances of death were grouped into bathing/swimming (a broader category that includes various water-related activities in different environments), fall from land (including bridges and jetties), boating (including fall from boat, raft/SUP or jetski), diving, ice related (including fall through ice by walking, ice skating or driving a vehicle such as a snowmobile), transport accident (driving a vehicle into water) and, bathtub (specific to activities occurring in a bathtub). Narcotics were grouped into opiates, central stimulants (amphetamine, methamphetamine, cocaine, methylenedioxymethamphetamine (MDMA)), benzodiazepines (including the benzodiazepine-like sedatives zopiclone and zolpidem) and tetrahydrocannabinol (THC). Drugs administered during resuscitation efforts were excluded.

### Ethics approval and consent to participate

No ethical approval was required for the current study, as stated by The Swedish ethical review board (decision date 15 August 2023 #2023–03922-01).

## Results

### General trends, sex and age

**Fig. 1:**
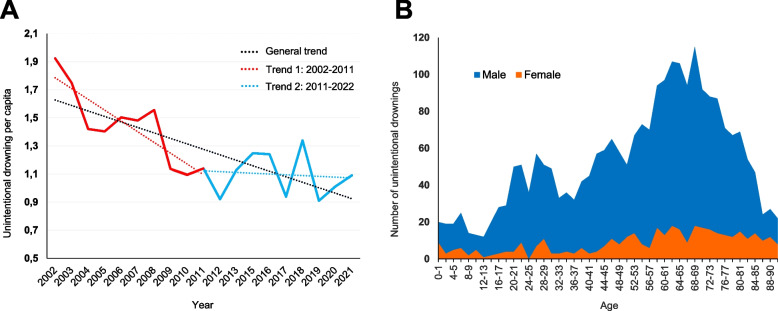
**A** Unintentional drowning deaths in Sweden between 2002 and 2021, incidence per 100 000. General trend demonstrates significant decline in drownings during total study period (*r* = -0. 0.779; *p* < 0.01). Multiple trendlines were also identified spanning two periods, trend 1: 2002 – 2011 (*r* = -0.836; *p* < 0.05) and trend 2: 2011 – 2021 (*r* = -0.172). **B** Unintentional drowning deaths 2002–2021, age and sex distribution

A total of 4420 drowning deaths occurred during the 20-year study period. 2443 (55%) were assessed as unintentional, 1321 (30%) suicide and 656 (15%) as undetermined (supplementary Table S1). There was a general decline in the number of unintentional drowning deaths between 2002 and 2021 (*r* = -0.779; *p* < 0.01). Piecewise regression analysis identified two different trends within the study period. Initially, unintentional drowning decreased (*r* = -0.836; *p* < 0.05) during the first nine years of the study period from 172 cases (1.9 per 100 000 inhabitants) in 2002 to 103 cases in 2011 (1.1 per 100 000 inhabitants), with a mean of 132 cases per year (1.4 per 100 000 inhabitants) (Fig. 1A). However, no significant trend was observed in drowning rates during the subsequent ten-year period between 2011–2021 (*r *= -0.172; *p* > 0.05), remaining stable with an average of 112 cases per year (1.1 per 100 000 inhabitants).

The distribution of age and sex demonstrates that males were overrepresented in all age groups, even for young children, with 72.3% in subjects aged 0–4 years and 76.0% aged 5–9 years (Fig. 1B). The largest male dominance was seen in the age group 30–39 with 90.1%. Over 50% of all unintentional drownings recorded were of individuals aged 50 and above.

### Circumstances of death and body of water

**Fig. 2:**
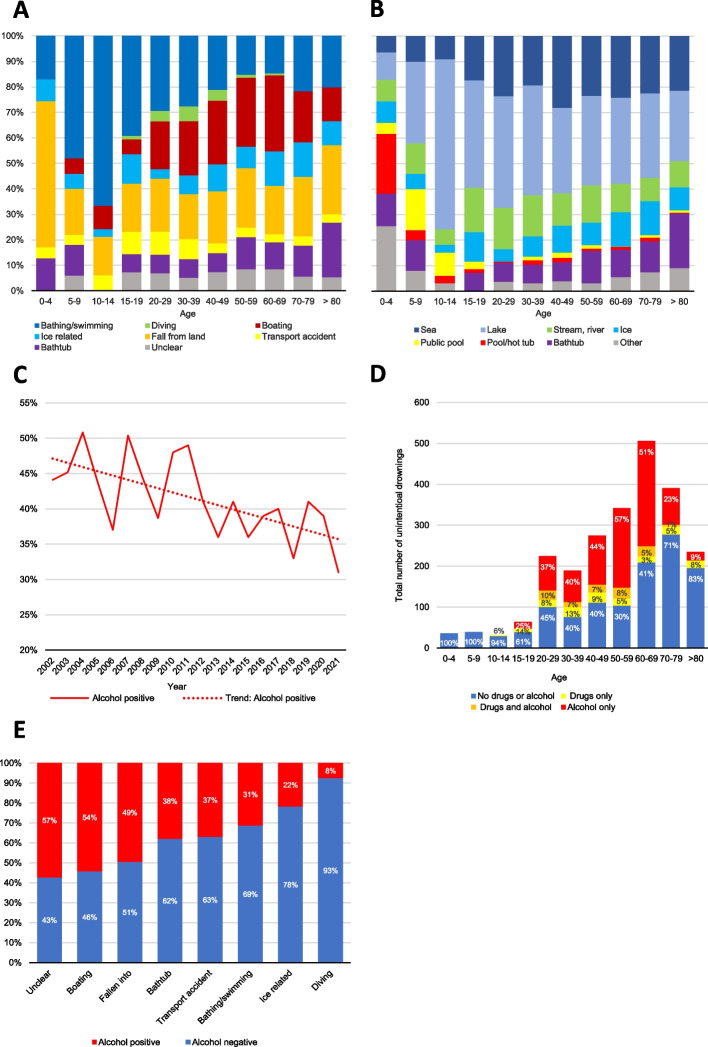
**A** Unintentional drowning deaths 2002–2021, activity and age distribution. **B** Unintentional drownings 2002–2021, body of water and age distribution. **C** Unintentional drowning deaths 2002–2021, presence of alcohol. BAC above 0.2 g/L in femoral blood at the time of autopsy were considered positive for alcohol. Trendline: decrease in alcohol positive cases study period (*r* = -0.695; *P* < 0.05). **D** Unintentional drownings 2002–2021, presence of alcohol and drugs grouped by age. **E** Alcohol positive and alcohol negative unintentional drownings between 2002–2021 by activity

Children aged 5–14 years drowned predominantly during bathing/swimming activities, while children aged 0–4 years mostly fall into water, often into private pools or shallow water (Fig. 2A and B). A large proportion of drowning deaths in the older age groups (40–49, 50–59, 60–69 and 70–79) occurred during boating activity or by falling into water from land (Supplementary Table S3).

### Alcohol and drugs

The proportion of alcohol positive cases decreased from 44% in 2002 to 31% in 2021 (*r* = -0.642; *p* < 0.05) (Fig. 2C). Results show that between 2010–2021, 31% of females and 41% of males exhibited a BAC of > 0.2 g/L and of those, 30% and 38%, respectively, exhibited a BAC of > 0.5 g/L (supplementary Table S2). During the entire study period (2002–2021), the largest proportion of alcohol positive cases was found in the age group 50–59 years (Fig. 2D), consisting of 55% females and 60% males (supplementary Table S2) Mean alcohol concentration was 1.85 g/L for females and 1.95 g/L for males.

Results indicate that the age group 30–39 years (25%) exhibited the greatest narcotic use (Fig. 2D). Regarding pre-drowning activity, the largest proportion of narcotics was found in the category “unclear” with 26% positive, followed by “bathtub” (17%) (data not shown). The categories with the largest proportion of alcohol positive cases were “boating” (54%) and “fall from land” (49%), while the categories with the least proportions were “ice-related” (22%) and “diving” (8%) (Fig. 2E).

### Region of origin

**Fig. 3:**
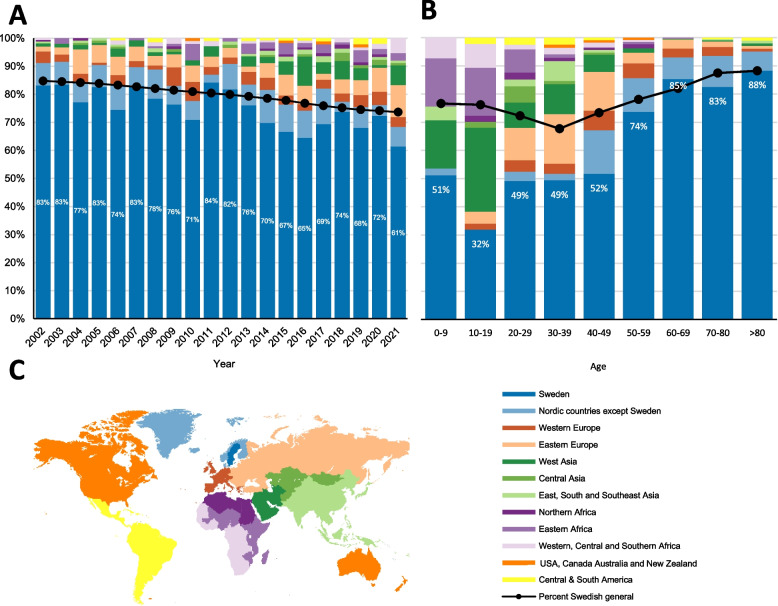
**A** Unintentional drownings 2002–2021, proportions of region of origin over time. **B** Unintentional drownings 2012–2021, region of birth and age groups. Proportion of persons defined as Swedish origin within the total Swedish population (official figures from SCB) is denoted by black dots/line. **C** County of origin grouped by region

In 2002, the Swedish population was approximately 9 million and 15% consisted of individuals of non-Swedish origin (Fig. 3A). In 2021, this figure had increased to 26% and the overall population had increased to 10.5 million, according to SCB. Considering the decrease in the proportion of individuals with Swedish origin in the national population, declining from 85% in 2002 to 74% in 2021, results demonstrate that individuals with non-Swedish origins faced a greater risk of unintentional drowning than those of Swedish origin (Table 1). This trend persisted across the 20-year period examined, with exceptions noted in 2007 where the risk was equal, and in 2011 and 2012, where the risk was higher for individuals with Swedish origin.

**Table 1:** Risk of unintentional drownings between 2002–2021, between persons with Swedish vs non-Swedish country of origin over time. 1 = risk of drowning is equal between Swedish and non-Swedish persons. > 1 = risk of drowning is greater for persons with country of origin not Sweden. < 1 = risk of drowning is greater for persons with Sweden as country of origin

Year	**2002**	**2003**	**2004**	**2005**	**2006**	**2007**	**2008**	**2009**	**2010**	**2011**	**2012**	**2013**	**2014**	**2015**	**2016**	**2017**	**2018**	**2019**	**2020**	**2021**
Swedish in total population (%)	85	85	84	84	84	83	82	81	81	80	80	79	79	78	77	76	75	75	74	74
Non-Swedish in total population (%)	15	15	16	16	16	17	18	19	19	20	20	21	21	22	23	24	25	25	26	26
Swedish in drowning population (%)	83	83	77	83	74	83	78	76	71	84	82	76	70	67	65	69	74	68	72	61
Non-Swedish in drowning population (%)	17	17	23	17	26	17	22	24	29	16	18	24	30	33	35	31	26	32	28	39
**Odds Ratio**	**1.16**	**1.16**	**1.57**	**1.08**	**1.84**	**1.00**	**1.28**	**1.35**	**1.74**	**0.76**	**0.88**	**1.19**	**1.61**	**1.75**	**1.80**	**1.42**	**1.05**	**1.41**	**1.11**	**1.82**

It is evident that individuals of non-Swedish origin are disproportionately more affected in the age range 0–49 (Fig. 3B). Data reveals a notable increase in the risk of drowning for non-Swedish individuals aged 0–9 with a ratio of 3.22. The highest risk was noted for non-Swedish origin individuals aged between 10–19, reaching a ratio of 6.73 (Table 2). Amongst these children, many were of West Asian and Eastern African descent, while in the age groups 30–49, a large number was of Eastern European origin.

**Table 2:** Risk of unintentional drownings between 2012–2021, between persons with Swedish vs non-Swedish region of origin in age groups. 1 = risk of drowning is equal between Swedish and non-Swedish persons. > 1 = risk of drowning is greater for persons with country of origin not Sweden. < 1 = risk of drowning is greater for persons with Sweden as country of origin

Age	**0–9**	**10- 19**	**20–29**	**30–39**	**40–49**	**50–59**	**60–69**	**70–80**	** > 80**
Swedish in total population (%)	77	76	72	68	73	78	82	87	88
Non-Swedish in total population (%)	23	24	28	32	27	22	18	13	12
Swedish in drowning population (%)	51	32	49	49	52	74	85	83	88
Non-Swedish in drowning population (%)	49	68	51	51	48	26	15	17	12
**Odds Ratio**	**3.22**	**6.73**	**2.68**	**2.21**	**2.50**	**1.25**	**0.80**	**1.37**	**1.00**

## Discussion

This study demonstrates firstly, that although unintentional drowning rates in Sweden have decreased between 2002 and 2021, they have plateaued since 2012. This finding is significant, indicating the need for an official national prevention strategy with annual evaluations. Secondly, results show that males are overrepresented in all ages, in line with previous findings [3, 11, 17, 18, 21], and that those aged ≥ 50 accounted for over half of all unintentional drownings by number. Thirdly, during the 20-year period (with the exception of 2007, 2011 and 2012), the risk of drowning was greater for non-Swedish individuals. In particular, non-Swedish persons below 20 years of age had a notably greater risk of unintentional drowning compared to Swedish individuals. It is evident that different populations are associated with greater or lesser risks of unintentional drownings, and that the variables examined and findings presented in this study should guide future preventative initiatives and evaluations in Sweden.

Consistent with prior research, results demonstrate a notable male predominance in drowning rates amongst subjects 0–4 years of age [3, 8, 9]. The notion that males exhibit greater exploratory and risk-taking behaviour compared to females has been previously discussed in areas of both medicine and psychology [22–24], and has been implicated to contribute to higher water/swimming confidence and potentially greater drowning rates [13, 21, 25, 26]. From a biological perspective, variations in the production of the hormone testosterone are suggested to partly account for such risk-taking behaviour [27]. However, testosterone alone is not responsible for the higher rates of drowning amongst 0–4-year-old boys, where production is not as pronounced [28]. Additionally, since the judgement centre of the male brain is not fully developed until age 25 [29], it is most likely that behavioural traits during infancy are influenced by a multitude of factors that require further consideration in relation to drowning. While the implementation of self-closing gates and fencing (at least 90 cm in height that cannot be entered from underneath), are effective strategies that can reduce risks for 0–4-year-olds [30], increased parental supervision is perhaps the single most influential factor to avoid unintentional drowning during early years.

A marked reduction in alcohol positive unintentional drownings was noted in our results during the study period. This may indicate an increased awareness regarding the risks of alcohol in proximity to water. Alternatively, the decrease may be fuelled by reduced drinking trends within the Swedish population, supported by the growing body of literature describing a worldwide decline in alcohol consumption, particularly amongst various religious groups and younger individuals [31, 32]. In line with this, no 10–14-year-olds and only 25% of 15–19-year-olds were deemed to have consumed alcohol prior to drowning in the current study. Research suggests that the rise in alcohol abstinence in young Swedish populations is not associated with demographic shifts itself [32], but rather parenting characteristics, including monitoring and restrictive cultural attitudes [33]. Consequently, the role of parenting characteristics in the context of unintentional drowning should be further investigated.

For individuals who did consume alcohol, a noteworthy proportion exhibited BAC levels surpassing 0.5 g/L. The age group 50–59 demonstrated the greatest risk of drowning under the influence of alcohol, with a mean BAC of 1.85 g/L for females and 1.95 g/L for males. Such elevated alcohol levels are more typically correlated with individuals experiencing alcohol dependence, as opposed to individuals who engage in moderate, less frequent alcohol consumption [34]. Interestingly, males aged between 30–39 exhibited the greatest narcotic use, and was in 17% of cases, identified as having drowned in bathtubs.

The circumstances surrounding unintentional drowning fatalities suggest that individuals in younger age groups (5–29 years) are more prone to drowning during bathing/swimming, with the majority occurring in lakes. Janson et al. [16], reported comparable results, proposing that inadequate parental supervision was a major contributing factor for younger children, whilst older children tended to overestimate their swimming abilities. Data concerning the use of life jackets from police reports is scarce, but it may be proposed that bathing/swimming activities should be conducted with life jackets if swimming competency is low, as organized lifeguarding services are only available on a number of established coastal beaches around the country and are less common at lakes. The establishment of designated swimming areas at such locations could potentially reduce drownings by concentrating bathing and swimming activities into populated zones [35]. This allows for increased supervision by bystanders, access to rescue equipment [36], and may facilitate prompt responses in cases of emergency.

In older age groups (30–69 years), fatalities related to boating activities were more prevalent (50% alcohol positive), and the demographic most susceptible to drowning while boating coincided with the age group that consumed the greatest volume of alcohol (50–69 years). Interestingly, Janson et al. [16] reported no drownings of children (< 17 years) in the context of boating activities, indicating a potential influence of parental vigilance in ensuring the utilization of life jackets and reduced alcohol consumption at sea when children were present. This trend did not continue in the current study, and may be due to adherence factors such as comfort, accessibility and ownership that may lead to decreased life jacket use [37]. Currently, there is no Swedish law mandating the use of life jackets for either adults or children on smaller vessels. However, in 2010, legislation was introduced to limit alcohol consumption at sea, aligning with drunk-driving limits and in 2022 additional legislation was enacted to regulate the operation of recreational watercraft, such as jet skis, without a driver's license. Future analyses should examine the impact of these regulations on drowning deaths related to boating, considering the role of alcohol consumption both before and after the implementation of these legislative actions. Naturally, preventive measures need to further address the risks associated with alcohol and boating, advocate the use of life jackets, as well as consider heightened maritime policing together with stringent legislation, as increased regulation regarding the use of life jackets during recreational boating has shown to decrease drowning in other countries [38].

The highest incidence of drowning following falls from land occurred amongst infants (0–5 years) and the elderly (> 80 years), who also faced an elevated risk of drowning in a bathtub that may have been triggered by a fall [39]. Bathtubs used by an elderly individual should be enhanced to incorporate additional safety features to mitigate potential risks of falling, in addition to increased supervision. Such preventative measures will become increasingly important due to an aging population. Furthermore, falling into water (sea, lakes, rivers, and through ice) in Sweden may carry a greater risk of activating the body’s cold shock response compared to warmer climate countries. The sudden immersion can result in rapid gasping for air, hyperventilation, and potential cardiac arrest that increases the likelihood of drowning [40]. Additionally, the extended duration of cold-water exposure may also exacerbate the risk of hypothermia, resulting in higher incidences of cold water-related fatalities.

The current study is the first to examine unintentional drowning in relation to origin within Sweden, though is not the first to suggest that non-Swedish children carry a significantly greater risk of unintentional drowning compared to Swedish individuals. Janson et al. [16] noted that twice as many children with Middle Eastern origin drowned between 1998–2007, compared to children with Swedish origin. This was particularly pronounced in children of African descent [16]. A similar trend was observed in the current study, where a large proportion (47%) of children were of Eastern African and West Asian descent. Conversely, in older age groups (20–29, 30–39 and 40–49) 12%, 18% and 14% were of Eastern European origin, respectively, and in age groups > 40, individuals of other Nordic countries were the largest populations observed after those of Swedish origin, reflecting migration patterns during the late twentieth century.

Both ethnic and racial disparities have been reported within American populations [8, 10, 11]. For instance, Clemens et al. [8] found that Indian/Alaskan Native and Black individuals have a higher drowning risk compared to White, Asian/Pacific Islander, and Hispanic individuals aged ≤ 29 in the US between 1999 and 2019. The examination of both race and ethnicity as a single variable might itself limit conclusions, as it may be argued that socio-economic status predominantly shapes racial disparities, while ethnic disparities are more influenced by differences in culture. Although a number of studies have examined unintentional drowning in Europe [14, 41], too few have examined ethnic disparities. Our results support previous findings of Bierens and Hoogenboezem [15], who also noted that unintentional drowning rates in persons with a non-western migration background is almost twice as high than for persons with a western migration or native Dutch background.

It may be hypothesized that migration from regions where exposure to recreational water activities is scarce, to regions where greater exposure increases risks primarily attributed to deficits in water competency. Sweden, as well as several other European countries, boasts abundant lakes and safeguarded coastal areas that encourage regular swimming and bathing activities from a young age. It may be postulated that amongst individuals of non-Swedish backgrounds, the heightened risk of drowning therefore stems from infrequent recreational exposure to water prior to migration, resulting in the inadequate water competency and limited swimming proficiency. Indeed, inadequate water competency need not be exclusive to individuals with non-Swedish backgrounds, but rather differences in geography that can also occur within the same country. Studies examining drowning following internal migration in China indicate that resettling populations are more vulnerable than those permanently residing in coastal areas [42]. Although Sweden and China face vastly different challenges in organizing national swimming competency campaigns due to varying traditions, political contexts, and geographic factors, it is crucial to acknowledge that local research is key to informing and shaping global policies.

Targeted interventions should consider accessible information in native languages that consist of swimming lessons, use of flotation devices and water competency. The establishment of designated swimming/bathing areas with accessible roads for emergency transportation should also be considered at popular lakes and coastal areas. Fostering water competency, not only swimming proficiency, is crucial, as swimming proficiency alone may not influence outcomes in drownings in bathtubs or during car and snowmobile accidents [16]. Further educational components should include risk perception, comprising information on the influences of alcohol and narcotics use as well as lifesaving and cardiopulmonary resuscitation (CPR).

### Study limitations

There are a number of study limitations that need to be considered. For instance, the NBFM database does not include comprehensive details regarding occupational or work-related drownings, complete details surrounding life jacket usage, victim native language, time in Sweden or religious beliefs. Such information is only available if explicitly mentioned in police reports and analysis was therefore excluded in this study.

Furthermore, while acknowledging the importance of geographical origins in identifying target demographics for public health campaigns, it is crucial to recognize that our approach considering region of origin is broad, governed by a political landscape, and may only serve as a proxy for cultural differences. This is because culture is not a measure of swimming capability or parental supervision. Arguably, neither is ethnicity, though understanding backgrounds, languages and traditions may provide insight into culture, socio-economic status, and education.

Additionally, the definition of individuals with Swedish origin may itself be a limiting factor that needs to be considered when interpreting results from this study. Our binary categorization could lead to oversimplification and misrepresentation overlooking first or second-generation migrants who have undergone Swedish education and lived in Sweden their entire lives. Future research on drowning risks specific to persons of non-Swedish origin is warranted to further identify vulnerable populations.

### Implications

Findings of the current study indicate that drowning prevention strategies should consider the following implications:

- Increased parental supervision for young children: Maintain close supervision, ensuring that children under the age of five are always within arm's reach.
- Life jacket usage for specific groups: Children and individuals lacking water competency should wear life jackets during bathing/ swimming. Life jackets should be worn at all times during boating, even when engine is not running.
- Securing water-related facilities and establishing designated swimming areas: This should include self-closing and self-latching gates.
- Alcohol and narcotics avoidance in proximity to water: Refrain from consuming either in the vicinity of water, especially during swimming and boating activities.
- Integration of water safety education: Implement swimming and water safety competency courses in schools and local communities. Ensure that education is provided in other ethnic languages if needed.

## Conclusions

While drowning rates have declined in Sweden between 2002–2021, this study reveals a plateau in unintentional drowning rates since 2012. The finding signals for an official national prevention strategy to reverse this trend. This includes enhanced parental supervision for young children, abstaining from alcohol while swimming and boating (in particular men > 40 years of age), and fostering a more realistic comprehensive acquisition of an expanded water competency across all age groups. Additionally, the heightened risk of drowning amongst non-Swedish individuals, particularly those < 20 years of age, suggests the importance of tailoring prevention efforts to specific populations. Finally, we highlight the need for a Swedish National Centre for Drowning Prevention, responsible for outlining a national strategy for drowning prevention, further research and annual evaluations of drowning statistics, in line with recommendations from the UN global drowning prevention resolution A/RES/75/273 adopted on 28 April 2021.

## Supplementary Information

Supplementary Material 1.

## Data Availability

Data will be available with publication on request to the authors (proposals can be directed to brita.zilg@ki.se). Data will be shared with researchers who provide a methodologically sound proposal.
