# Supplementary material for: Unintentional drowning fatalities in Sweden between 2002 and 2021
Source: BMC Public Health. 2024 Nov 16;24:3185. doi: 10.1186/s12889-024-20687-3 (PMC11568521; doi:10.1186/s12889-024-20687-3)
Supplement: Supplementary file 1 — Supplementary Material 1. [file 12889_2024_20687_MOESM1_ESM.docx]

**Supplementary Material**

**Table S1.**

| **Year** | **Unintentional** | | |  | **Suicide** | | |  | **Undetermined** | | |  | **Total** |
| --- | --- | --- | --- | --- | --- | --- | --- | --- | --- | --- | --- | --- | --- |
|  | **Female** | **Male** | **Total** |  | **Female** | **Male** | **Total** |  | **Female** | **Male** | **Total** |  |  |
| **2002** | 29 | 143 | **172** |  | 44 | 44 | **88** |  | 16 | 31 | **47** |  | **307** |
| **2003** | 24 | 133 | **157** |  | 35 | 27 | **62** |  | 11 | 29 | **40** |  | **259** |
| **2004** | 21 | 107 | **128** |  | 38 | 33 | **71** |  | 13 | 22 | **35** |  | **234** |
| **2005** | 17 | 110 | **127** |  | 44 | 32 | **76** |  | 10 | 22 | **32** |  | **235** |
| **2006** | 21 | 116 | **137** |  | 36 | 42 | **78** |  | 11 | 35 | **46** |  | **261** |
| **2007** | 19 | 117 | **136** |  | 35 | 31 | **66** |  | 10 | 27 | **37** |  | **239** |
| **2008** | 27 | 117 | **144** |  | 25 | 41 | **66** |  | 9 | 37 | **46** |  | **256** |
| **2009** | 12 | 94 | **106** |  | 36 | 39 | **75** |  | 12 | 21 | **33** |  | **214** |
| **2010** | 25 | 78 | **103** |  | 21 | 31 | **52** |  | 6 | 13 | **19** |  | **174** |
| **2011** | 19 | 89 | **108** |  | 32 | 24 | **56** |  | 10 | 21 | **31** |  | **195** |
| **2012** | 11 | 77 | **88** |  | 30 | 34 | **64** |  | 8 | 24 | **32** |  | **184** |
| **2013** | 14 | 95 | **109** |  | 37 | 44 | **81** |  | 12 | 21 | **33** |  | **223** |
| **2014** | 21 | 115 | **136** |  | 40 | 27 | **67** |  | 12 | 18 | **30** |  | **233** |
| **2015** | 25 | 98 | **123** |  | 38 | 38 | **76** |  | 15 | 28 | **43** |  | **242** |
| **2016** | 18 | 106 | **124** |  | 35 | 27 | **62** |  | 9 | 19 | **28** |  | **214** |
| **2017** | 16 | 79 | **95** |  | 29 | 28 | **57** |  | 7 | 17 | **24** |  | **176** |
| **2018** | 20 | 117 | **137** |  | 29 | 42 | **71** |  | 3 | 26 | **29** |  | **237** |
| **2019** | 21 | 73 | **94** |  | 15 | 25 | **40** |  | 5 | 24 | **29** |  | **163** |
| **2020** | 21 | 84 | **105** |  | 21 | 36 | **57** |  | 5 | 14 | **19** |  | **181** |
| **2021** | 15 | 99 | **114** |  | 25 | 31 | **56** |  | 9 | 14 | **23** |  | **193** |
| **Total** | **396** | **2047** | **2443** |  | **645** | **676** | **1321** |  | **193** | **463** | **656** |  | **4420** |

Drowning deaths by year, sex, and manner of death.

**Table S2.**

|  | **Alcohol positive  (>0,02 g/L)** | | | | **Alcohol positive  (>0,05 g/L)** | | | | **BAC median, g/L** | | **Drugs positive** | | | |  |
| --- | --- | --- | --- | --- | --- | --- | --- | --- | --- | --- | --- | --- | --- | --- | --- |
| **Age** | **f** | | **m** | | **f** | | **m** | | **f** | **m** | **f** | | **m** | |  |
|  | **n** | **%** | **n** | **%** | **n** | **%** | **n** | **%** |  |  | **n** | **%** | **n** | **%** | |
| 0-9 | 0 | 0% | 0 | 0% | 0 | 0% | 0 | 0% | 0 | 0 | 0 | 0% | 0 | 0% | |
| 10-19 | 0 | 0% | 6 | 12% | 0 | 0% | 5 | 10% | 0 | 1.34 | 0 | 0% | 8 | 13% | |
| 20-29 | 6 | 40% | 59 | 53% | 5 | 33% | 51 | 46% | 1.90 | 1.63 | 5 | 3% | 22 | 15% | |
| 30-39 | 2 | 25% | 38 | 44% | 2 | 25% | 32 | 37% | 0.96 | 1.95 | 5 | 5% | 20 | 20% | |
| 40-49 | 6 | 38% | 57 | 49% | 6 | 38% | 55 | 47% | 1.97 | 2.07 | 1 | 1% | 21 | 15% | |
| 50-59 | 12 | 55% | 75 | 60% | 12 | 55% | 72 | 58% | 1.77 | 2.19 | 5 | 3% | 23 | 14% | |
| 60-69 | 23 | 52% | 136 | 55% | 23 | 52% | 130 | 52% | 2.10 | 2.11 | 7 | 2% | 20 | 7% | |
| 70-79 | 11 | 26% | 41 | 23% | 11 | 26% | 40 | 22% | 1.62 | 1.45 | 3 | 1% | 7 | 3% | |
| 80-90 | 3 | 8% | 10 | 11% | 1 | 3% | 7 | 8% | 0.35 | 0.38 | 3 | 2% | 8 | 6% | |
| >90 | 0 | 0% | 1 | 13% | 0 | 0% | 0 | 0% | - | 0.36 | 1 | 8% | 1 | 8% | |
| **Total** | **63** | **31%** | **423** | **41%** | **60** | **30%** | **392** | **38%** | **1.85** | **1.95** | **30** | 2% | **130** | **10%** | |

Unintentional drownings, alcohol and drugs, 2010-2021. Proportions between sexes and age groups of those tested for alcohol. BAC = blood alcohol concentration.

**Table S3.**

|  | **Bathing/ swimming** | | **Bathtub** | | **Boating** | | **Diving** | | **Fallen into** | | **Ice related** | | **Transport accident** | | **Unclear** | | **Total** |
| --- | --- | --- | --- | --- | --- | --- | --- | --- | --- | --- | --- | --- | --- | --- | --- | --- | --- |
| **Age** | **f/m** | **total** | **f/m** | **total** | **f/m** | **total** | **f/m** | **total** | **f/m** | **total** | **f/m** | **total** | **f/m** | **total** | **f/m** | **total** |  |
| **0-4** | 5/3 | 8 | 1/5 | 6 | 0/0 | 0 | 0/0 | 0 | 7/20 | 27 | 0/4 | 4 | 0/2 | 2 | 0/0 | 0 | **47** |
| **5-9** | 5/19 | 24 | 3/3 | 6 | 0/3 | 3 | 0/0 | 0 | 2/7 | 9 | 1/2 | 3 | 1/1 | 2 | 0/3 | 3 | **50** |
| **10-14** | 4/18 | 22 | 0/0 | 0 | 1/2 | 3 | 0/0 | 0 | 1/4 | 5 | 0/1 | 1 | 0/2 | 2 | 0/0 | 0 | **33** |
| **15-19** | 2/25 | 27 | 3/2 | 5 | 1/3 | 4 | 1/0 | 1 | 2/11 | 13 | 0/8 | 8 | 0/6 | 6 | 0/5 | 5 | **69** |
| **20-29** | 6/66 | 72 | 10/8 | 18 | 1/45 | 46 | 3/7 | 10 | 3/48 | 51 | 0/9 | 9 | 6/16 | 22 | 2/15 | 17 | **245** |
| **30-39** | 3/50 | 53 | 10/4 | 14 | 1/40 | 41 | 1/10 | 11 | 2/32 | 34 | 1/13 | 14 | 0/15 | 15 | 1/9 | 10 | **192** |
| **40-49** | 7/53 | 60 | 9/12 | 21 | 7/64 | 71 | 3/9 | 12 | 2/56 | 58 | 3/27 | 30 | 0/11 | 11 | 2/19 | 21 | **284** |
| **50-59** | 9/45 | 54 | 20/25 | 45 | 8/88 | 96 | 1/3 | 4 | 12/71 | 83 | 3/27 | 30 | 1/12 | 13 | 3/27 | 30 | **355** |
| **60-69** | 23/53 | 76 | 23/32 | 55 | 6/149 | 155 | 0/4 | 4 | 11/88 | 99 | 5/65 | 70 | 1/15 | 16 | 5/39 | 44 | **519** |
| **70-79** | 24/64 | 88 | 18/31 | 49 | 4/77 | 81 | 0/0 | 0 | 15/79 | 94 | 2/53 | 55 | 5/10 | 15 | 4/19 | 23 | **405** |
| **80-90** | 15/31 | 46 | 27/15 | 42 | 1/31 | 32 | 0/0 | 0 | 12/49 | 61 | 3/19 | 22 | 2/5 | 7 | 2/9 | 11 | **221** |
| **>90** | 0/3 | 3 | 6/4 | 10 | 0/0 | 0 | 0/0 | 0 | 1/4 | 5 | 0/1 | 1 | 0/1 | 1 | 1/1 | 2 | **22** |
| **Total** | **103/430** | **533** | **130/141** | **271** | **30/502** | **532** | **9/33** | **42** | **70/469** | **539** | **18/229** | **247** | **16/96** | **112** | **20/146** | **166** | **2442** |

Unintentional drownings 2002-2021, age/sex distribution and activity. (f/m=female/male)
